# A Multi-Oscillatory Circadian System Times Female Reproduction

**DOI:** 10.3389/fendo.2015.00157

**Published:** 2015-10-20

**Authors:** Valérie Simonneaux, Thibault Bahougne

**Affiliations:** ^1^Institut des Neurosciences Cellulaires et Intégratives, CNRS (UPR 3212), Strasbourg, France; ^2^Service d’Endocrinologie et Diabète, Hôpital Civil, Hôpitaux Universitaires de Strasbourg, Strasbourg, France

**Keywords:** female reproduction, circadian clock, suprachiasmatic nuclei, kisspeptin, GnRH, LH, estradiol, shift-work

## Abstract

Rhythms in female reproduction are critical to insure that timing of ovulation coincides with oocyte maturation and optimal sexual arousal. This fine tuning of female reproduction involves both the estradiol feedback as an indicator of oocyte maturation, and the master circadian clock of the suprachiasmatic nuclei (SCN) as an indicator of the time of the day. Herein, we are providing an overview of the state of knowledge regarding the differential inhibitory and stimulatory effects of estradiol at different stages of the reproductive axis, and the mechanisms through which the two main neurotransmitters of the SCN, arginine vasopressin, and vasoactive intestinal peptide, convey daily time cues to the reproductive axis. In addition, we will report the most recent findings on the putative functions of peripheral clocks located throughout the reproductive axis [kisspeptin (Kp) neurons, gonadotropin-releasing hormone neurons, gonadotropic cells, the ovary, and the uterus]. This review will point to the critical position of the Kp neurons of the anteroventral periventricular nucleus, which integrate both the stimulatory estradiol signal, and the daily arginine vasopressinergic signal, while displaying a circadian clock. Finally, given the critical role of the light/dark cycle in the synchronization of female reproduction, we will discuss the impact of circadian disruptions observed during shift-work conditions on female reproductive performance and fertility in both animal model and humans.

## Introduction

Ovulation in female mammals is a complex process, which is exquisitely regulated by a number of environmental (time of day, time of year, food resources, and stress level) and internal (development stage, hormonal milieu, and metabolic rate) factors. Indeed, female reproduction is a long-term, demanding process and therefore, it is important that a limiting critical status is reached to ensure successful reproductive outcome. In adult females where all these criteria are attained, there are still two important cues that time ovulation: the circulating level of gonadal hormones, specifically estradiol, which is an indicator of oocyte maturation, and the time of day arising from biological clocks. This dual regulation ensures that the timing of ovulation coincides with the period of maximal activity and sexual motivation. Most mechanistic studies aimed at understanding this subtle timing of ovulation have been performed in laboratory rodents, but ovulation in humans is also gated by similar hormonal and circadian inputs. Hence, this review will not only focus on the mechanisms regulating the timing of reproduction in female rodents, but will also discuss human female fertility, including the desynchronization associated with modern life styles (shift work, jet lag, and sleep alteration).

## Female Reproduction is Rhythmic

Reproductive activity in female mammals displays a regular cycle (menstrual cycles in women, estrous cycles in rodents) driven by a complex interaction of the circadian system, hypothalamic neuropeptides, gonadotropins [luteinizing hormone (LH) and follicle-stimulating hormone (FSH), both secreted by the pituitary gonadotroph cells], and sex steroid hormones produced by the ovaries. The final output of this regulatory process is to combine the production of a mature oocyte (ovulation) with a receptive reproductive tract, which will ensure the embryo’s development.

**Figure 1:**
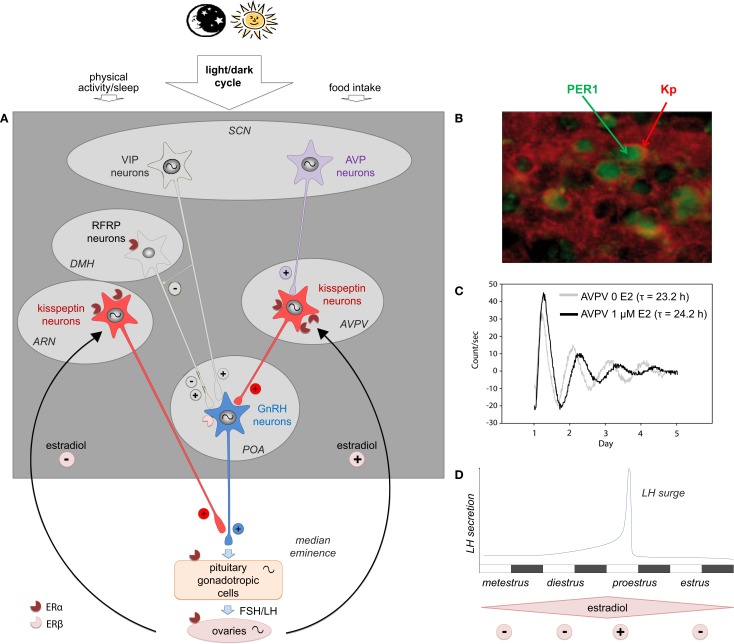
**The multi-oscillatory network of the female hypothalamic–pituitary gonadal axis in rodents**. **(A)** Schematic representation of the neuroendocrine pathway timing female reproduction: the master circadian clock located in the hypothalamic suprachiasmatic nucleus (SCN) is synchronized to the daily cycle mostly via the light/dark cycle and to a less extend by other time cues (food intake and sleep/wake activity); two SCN peptidergic transmitters forward the daily information to the reproductive axis: arginine vasopressin (AVP) is the most essential daily transmitter projecting to the kisspeptin (Kp) neurons of the anteroventral periventricular nuclei (AVPV), which in turn strongly activate the GnRH neurons located in the preoptic area (POA); vasoactive intestinal peptide (VIP) directly modulates GnRH neuron activity and possibly indirectly via neurons of the dorsomedial hypothalamus (DMH), which express RF-related peptide (RFRP); GnRH released in the portal blood system activates the synthesis and release of the gonadotropins luteinizing hormone (LH) and folliculo-stimulating hormone (FSH), which in turn regulate oocyte maturation, estradiol synthesis, and finally ovulation triggered by the LH surge; throughout the estrous cycle, estradiol feeds back onto different levels of the reproductive axis principally via the nuclear estrogen receptor α (ERα) and to a less extend via ERβ. Kp neurons are the main estradiol targets with an inhibitory effect of low estradiol on Kp neurons of the arcuate nucleus (ARN) and a stimulatory action of high estradiol on AVPV Kp neurons. In addition to the master SCN clock, peripheral clocks are located in the AVPV Kp neurons, GnRH neurons, pituitary gonadotrophs, and the different cell types of the ovary. **(B)** Representative pictures showing PER1 immunoreactivity (green) in Kp immunoreactive neurons (red) in the AVPV of female mice sampled at zeitgeber time 20 (8 h after lights off on a 12 h light/12 h dark schedule) on the day of proestrus [adapted from Ref. (9)]. **(C)** Representative bioluminescence traces from isolated AVPV explants of PER2:LUCIFERASE female mice, in absence (0 E2, light gray line) or in presence of estradiol (1 μM E2, black line) in the culture medium; τ indicates the period of the circadian AVPV oscillations in both conditions [adapted from Ref. (9)]. **(D)** Schematic representation of the mouse estrus cycle including the four stages: metestrus, diestrus, proestrus (the stage at which the LH concentration raises as an LH surge), and estrus; during the first part of the reproductive cycle, low circulating level of estradiol displays a negative feedback, whereas at proestrus, high estradiol levels exert a positive feedback, causing a synchronized activation of GnRH neurons leading to the preovulatory surge of LH; notably, the LH surge occurs at the end of the resting period, which is late day in nocturnal species.

During the first part of the reproductive cycle (follicular phase in women; metestrus–diestrus in rodents), gonadotrophs produce more FSH than LH. This relative FSH preponderance contributes to the recruitment and development of ovarian follicles. FSH promotes follicular growth leading to a progressive increase of the sex steroid hormone, estradiol, and increases LH receptor expression in granulosa cells (1). During this early phase, LH pulses occur with a high frequency (period of 1–2 h in women, 20 min in rodents) and uniform amplitude, and the pulse frequency tends to increase toward the end of the phase. The second part of the reproductive cycle (luteal phase in women; proestrus–estrus in rodents) begins with a marked and transient secretion of LH (Figures 1A,D). The LH surge has three functions: (1) induction of ovulation of mature follicles, (2) resumption of oocyte meiosis, and (3) arrest of granulosa cell proliferation and luteum induction. After the LH surge, ovulation generally follows a few hours later in rodents, and 24–48 h in women. The preovulatory LH surge takes place approximately every 4–5 days in rodents and every 28 days in women and its occurrence depends on high circulating estradiol levels (2, 3). Additionally, the LH surge requires a daily signal since it arises at a very specific time of day, usually at the end of the resting period, thus in the late afternoon in nocturnal rodents and the end of the night/early morning in the diurnal rodent Arvicanthis (4) and in humans (5–7). Indeed, in 80% of women, the LH surge occurs around 8 a.m. At the end of the reproductive cycle, LH pulse frequency decreases significantly down to a pulse interval of 2–6 h with variable amplitude (8).

The secretion of both LH and FSH is under the control of a hypothalamic neurohormone, gonadotropin-releasing hormone (GnRH), which is synthesized in neurons scattered throughout the preoptic area (POA) and the organum vasculosum laminae terminalis. These neurons project to the median eminence where they release GnRH in the portal circulation in a pulsatile manner. GnRH activates specific receptors located on pituitary gonadotrophs inducing the synthesis and release of LH and FSH. GnRH is essential for reproduction as mutations in the gene coding for GnRH (10) and GnRH receptor (11) are proposed to be responsible for idiopathic hypogonadotropic hypogonadism (IHH), characterized by delayed puberty and infertility (11). The pulsatility of GnRH release is critical to induce proper gonadotropin secretion, and there is a tight correlation between GnRH and LH pulsatilities. Pulsatile administration of exogenous GnRH (one pulse per hour) is capable of restoring the preovulatory surge, ovulation, and normal menstrual cycles in patients suffering from Kallmann syndrome (12, 13). In contrast, continuous GnRH administration induces a reversible blockage of the pituitary gonadotroph cells’ secretion (14).

Various (neuro)transmitters had been proposed to regulate GnRH neuronal activity until the neuropeptide kisspeptin (Kp) was discovered as a potent activator of GnRH release. In 1996, the *Kiss1* gene was discovered and reported to encode a peptide called metastin, because of its anti-metastatic property on malignant melanoma cells (15). However, the receptor of this peptide, GPR54, was later found to play a critical role in reproductive physiology when two groups reported that mutation of the *GPR54* receptor results in IHH in humans, with an identical phenotype observed in mice with a targeted deletion in this receptor (16, 17). The *Kiss1* gene was shown to encode a family of Kps from an initial 145 amino acid propeptide, Kp-145, which is cleaved into peptides of different sizes from Kp-54 (previously named metastin) to Kp-10. The discovery of Kp’s role in reproductive function has been a milestone in the field of reproductive biology, and numerous studies now indicate that Kps are critical regulators of sexual differentiation and maturation as well as of normal adult reproductive functioning across mammalian species, including humans (18). Kp neurons are localized within two hypothalamic areas, in the arcuate nucleus (ARN) and the rostral periventricular nucleus of the third ventricle, also called anteroventral periventricular nucleus (AVPV), or the preotic area (according to species). They send projections mainly to the GnRH neuron cell bodies (AVPV Kp neurons) and nerve terminals [ARN Kp neurons (19–22)] (Figure 1A). The AVPV presents a marked sexual dimorphism, with more Kp neurons in females as compared to males (20, 23). The AVPV Kp neurons are the main drivers of the preovulatory GnRH/LH surge (24). In contrast, the ARN Kp neurons are not sexually dimorphic (20, 23). The Kp receptor, Kiss1R (formerly GPR54), is highly expressed in GnRH neurons but also in other brain areas (25, 26) and in most endocrine tissues like the pituitary gland, ovary, and placenta (27). Kp has a very potent stimulatory action on GnRH release and, therefore, gonadotropin secretion in all mammalian species investigated so far (18, 19, 28, 29). Central injection of doses as low as 0.1–1 pmol Kp10 is indeed sufficient to evoke robust LH secretion in rats and monkeys (28, 30). Kp injections must be short and at least 2 h apart to induce the LH peak since the repeated administration of Kp induces Kiss1R desensitization (31, 32). Notably, Kp release in the stalk-median eminence is pulsatile (33), and pulsatile Kp drives LH secretion in juvenile monkeys (34). A recent study reported that pulsatile administration of Kp was able to evoke dramatic synchronous activation of *GnRH* gene transcription with robust stimulation of GnRH secretion in murine-cultured hypothalamic explants (35). The preeminent phenotypes of impaired reproduction (abnormal sexual maturation, small uterus, ovaries without mature follicles, no estrous cycles) often arise from mutations in *Kiss1* (36, 37) and *Kiss1R* (16, 38, 39), which suggest that the Kiss1/Kiss1R complex is essential for the central regulation of the gonadotropic axis.

Other classical neurotransmitters and neuropeptides have been reported to regulate GnRH neuron activity albeit not to the same extent as Kp. GABA and glutamate fibers are found close to GnRH perikarya in the POA and axons in the median eminence. Both neurotransmitters have been shown to play a role in the regulation of GnRH release. Glutamate stimulates *Gnrh* gene expression and GnRH release during the LH surge, whereas a glutamate antagonist blocks *Gnrh* gene expression and the LH surge when administered in the morning (40–42). Administration of an AMPA agonist enhances the *in vivo* LH secretion in OVX rats only with estradiol substitution, whereas glutamate stimulates *in vitro* GnRH secretion in a estradiol-independent matter (43). The role of GABA on GnRH neuronal activity is debated since inhibitory and stimulatory effects have been observed depending on the protocols used, the presence of sex steroid treatment, the timing in the estrus cycle and the hypothalamic region studied (44, 45). Fibers containing the orexigenic neuropeptide Y contact a majority of GnRH neurons, which express neuropeptide Y receptors. This neuropeptide has been reported to exert variable effects depending on the metabolic and reproductive status of the animal, but most of the studies describe an inhibitory effect of neuropeptide Y on GnRH neurons (46–48). Recent studies indicate that another neuropeptide belonging to the same RF-amide peptide family as Kp, RFRP-3 (the mammalian homolog of avian gonadotropin-inhibitory hormone), regulates GnRH neuron activity [for review, see Ref. (49–51)]. Unlike Kp, RFRP-3 can activate or inhibit the reproductive axis according to gender, species, and environmental conditions (26, 51–53). In female rodents RFRP neuronal activity is decreased at the time of the LH surge, possibly to relieve the inhibitory effect of RFRP-3 on GnRH neurons (54). However, mice bearing a null mutation of GPR147, the RFRP-3 receptor, present only a mild reproductive phenotype (55).

## Estrogenic Regulation

Estradiol produced by the ovaries exerts both positive and negative feedback upstream of the reproductive axis, modulating GnRH neuron activity and anterior pituitary gonadotroph cells. During the first part of the reproductive cycle, estradiol induces a negative feedback, whereas when estradiol concentration is the highest (at the end of the follicular phase in humans or proestrus in rodents) the feedback becomes positive, causing a synchronized activation of GnRH neurons leading to GnRH release in the hypophyseal portal blood and finally the preovulatory LH surge (56) (Figures 1A,D). The effect of estradiol is mediated via two types of nuclear estrogen receptors (ERs), which induce long lasting genomic action, ERα and ERβ (57, 58), but it can also have a rapid action via membrane bound estradiol receptors, including the GPR30 (59, 60).

### GnRH neurons

Gonadotropin-releasing hormone neurons contain few, if any, ERα (61–63), but do express ERβ (64–66) and GPR30 (67). Estradiol application to cultured primate GnRH neurons induces a rapid increase in action potential firing frequency (68) and intracellular calcium oscillations (69). Similar effects have been reported in the mouse GnRH neurons (70). This rapid effect of estradiol is proposed to be mediated via GPR30 in primates (67) and ERβ in mice (70, 71). Using an *in vitro* GnRH neuronal model, the GT1-7 cells, it was reported that *Kiss1R* expression is estradiol dependant, with a Kp-induced GnRH increase only in cells treated with estradiol (72, 73). A primary effect of estradiol on GnRH neurons has been hypothesized to upregulate expression of channel transcripts (TRPC4 channels and HCN1 channels) that orchestrate the downstream signaling of Kiss1R in GnRH neurons (74, 75). Therefore, estradiol could be a potent regulator of ion channel and receptor expression in GnRH cells, hence controlling the sensitivity of GnRH neurons to Kp. However, a recent study reported that mutant mice with a GnRH neuron-selective deletion of ERβ exhibit normal cycles and negative feedback, leaving the critical role for ERβ in GnRH neuron activity still an open question (76).

### Kisspeptin neurons

In contrast to GnRH, Kp neurons have a high density of ERα and are, therefore, considered as the intermediate node for the estradiol feedback on GnRH neurons (19, 20, 32, 61, 77–79) (Figure 1A). Interestingly, the estradiol feedback in rodents depends on the Kp neuron localization as estradiol stimulates *Kiss1* expression in the AVPV and inhibits *Kiss1* expression in the ARN (77, 80–82). In non-rodent mammals, a similar differential regulation by estradiol is also observed with a stimulatory effect in the rostral periventricular/POA and an inhibitory effect in the ARN (83, 84). The mechanism underlying these differences in the regulation of *Kiss1* expression by ERα in both structures is not yet fully understood. AVPV *Kiss1* activation requires an estrogen response element (ERE)-dependent pathway, whereas inhibition of *Kiss1* expression in the ARN involves ERE-independent mechanisms (80, 85). Recent studies have reported that estradiol additionally modulates daily activity of AVPV Kp neurons. Hence, the daily variation in c-Fos activation, *Kiss1* mRNA and peptide content observed in proestrus or in ovariectomized estradiol-treated rodents is abolished or strongly reduced in diestrus or in ovariectomized animals (9, 86–88). Furthermore, estradiol has been reported to increase the number of arginine vasopressin (AVP) synaptic contacts onto Kp neurons (89), regulate AVP_1a_ receptor expression by Kp neurons (88) and be permissive for the AVP-induced electrical activation of Kp neurons (90). Altogether these observations indicate that high circulating levels of estradiol gate the action of AVP onto AVPV Kp neurons (see Role of the Suprachiasmatic Nuclei for the role of AVPV on Kp neurons).

### Circadian system

Estradiol is also suggested to influence daily functions since shifts are observed during the pubertal period, pregnancy, menopause, and throughout the reproductive cycle (91–93). In rodents, ERα and ERβ are expressed in the retina, the retino-hypothalamic tract, the geniculohypothalamic tract and raphe nuclei-derived serotonergic inputs, all major inputs to the suprachiasmatic nuclei (SCN), which contain the master circadian clock (94). In mice, the second half of the proestrus night is often, but not consistently, characterized by increased motor activity compared to the remaining nights of the estrous cycle (95, 96). Furthermore, ovariectomy reduces total motor activity, and estradiol reverses this effect, while also shortening the length of the free running period and advancing the onset of wheel running activity (96). Estradiol may act directly on SCN clock gene oscillation since estradiol treatment in ovariectomized rats decreased *Cry2* mRNA levels (97) and estradiol application to SCN slices increased the spontaneous firing frequency and depolarized cell membranes of the SCN neurons (98). However, other studies reported that estradiol treatment of SCN explants from PER2:LUCIFERASE mice has no effect on the period and amplitude of the circadian oscillations (9, 99). Alternatively, estradiol may alter rhythms in running activity via indirect effects on the medial POA or striatum (100, 101).

### Pituitary

Pituitary gonadotrophs express ERα, and estradiol has been proposed to exert a direct negative feedback effect on gonadotropin secretion (102–104). Chronic treatment with estradiol induces negative feedback effects on gonadotrophin secretion after GnRH supraphysiologic stimulation in ewes (105) or humans (106). Interestingly, a recent study reported that mice with a selective deletion of ERα in pituitary gonadotroph cells had elevated serum LH and estradiol values, and displayed irregular estrous cycles punctuated by prolonged periods of disorganized cycling, pointing to ERα participation in the estradiol negative feedback at the pituitary level (107). It is worth mentioning that this phenotype was much less severe than the one observed after a total ERα deletion. Although the role of Kiss/Kiss1R at the pituitary is still the subject of debate, it is interesting to note that the activation of pituitary ERα up-regulates *Kiss1* expression, whereas chronic exposure to estradiol down regulates *Kiss1R* expression on pituitary gonadotrophs (108).

### Uterus

The uterus expresses high levels of ERα, and is an important site for the estrogenic control of reproductive physiology (109, 110). Estradiol, together with progesterone, regulates uterine growth and differentiation, which in turn control embryo-endometrial interactions during early pregnancy (110). Furthermore, estradiol treatment also shortens the period of the circadian clock in the uterus (99).

## Daily and Circadian Regulations

### Role of the suprachiasmatic nuclei

#### The Hypothalamic Suprachiasmatic Nuclei Host the Master Biological Clock

Most biological functions, including female reproduction, are synchronized to the daily variation of environmental factors. Among these factors, the recurring light/dark cycle is the most predictable environmental cue used by mammals to adjust their behavior and physiology appropriately. The mechanisms by which light and dark synchronize biological functions involve the master biological clock located in the hypothalamic SCN and a retino-hypothalamic tract, which forwards the non-visual light to the SCN.

The demonstration that a biological clock located in the basal hypothalamus was driving circadian rhythms came from experiments showing that SCN lesions in rodents abolished circadian rhythms in locomotor activity, which were restored following exogenous SCN implants (111, 112). The circadian activity of SCN neurons relies on a complex molecular system cycling endogenously with a period of about 1 day (*circa dies*). This molecular clockwork is composed of transcription–translation loops, which are now well described (113, 114). Dimers of the CLOCK and BMAL1 proteins bind to a specific E-box promoter to induce the transcription of four clock genes *Per1*, *Per2*, *Cry1*, and *Cry2*, which after translation produce proteins which form dimers to repress their own transcription by competing with the CLOCK/BMAL1 binding. Following degradation of the inhibitory proteins, the transcription–translation loop starts over for another circadian cycle. When SCN explants or dissociated cells are kept *in vitro*, the endogenous circadian oscillations continue for weeks or months, providing that the culture medium gets enough nutrients and oxygen for the cell metabolism. A very interesting animal model used to demonstrate these sustained endogenous oscillations is the PER2:LUCIFERASE mice, where the expression of the *luciferase* gene is driven by the *Per2* promoter (115, 116). When explants or dissociated cells of PER2:LUCIFERASE mouse SCN are placed in a culture medium containing luciferine, the rhythmic expression of PER2 drives a rhythmic expression of luciferase, which by oxidizing luciferin causes the emission of a bioluminescent signal with a circadian period.

In order to achieve its role in adjusting biological functions with the astronomical daily cycles, the SCN circadian activity has to be synchronized with the time of day and transmit this timing information to the rest of the body. Light has long been known to be the main synchronizer of the SCN circadian clock, but interestingly it uses a specific non-visual pathway, which includes melanopsin-containing intrinsically photosensitive retinal ganglion cells projecting directly to the SCN (117, 118). Upon light activation, these ganglion cell terminals release glutamate and pituitary activating cAMP peptide, which change the phase (synchronizes) of the circadian clock. The synchronizing property of light depends on the time of application during the day, and the characteristics of the phase responses depend on species. The astronomical light/dark alternation synchronizes the circadian clock in order to attain a daily rhythm of a precise 24-h period. The CLOCK/BMAL1 dimers not only activate canonical clock gene expression, but other clock-controlled genes whose promoters display E-boxes and therefore undergo rhythmic expression. This mechanism was first demonstrated for the gene encoding AVP, an important output of the SCN clock (119). Levels of SCN AVP mRNA are markedly higher during the day than at night, but in *Clock* KO mice the SCN AVP rhythm is strongly dampened (120).

#### SCN Lesion or Clock Gene Mutations Alter the Reproductive Cycle

Various experiments aiming at impairing clock function were performed to delineate whether functional SCN neurons are required for the daily timing of the LH surge, mostly in female rodents. Early experiments of SCN lesions (121) or SCN-POA neuronal connection cut (122) resulted in an impaired LH surge and estrous cyclicity in female rats. Furthermore, female mice carrying mutations of *Clock* or *Bmal1* displayed disrupted estrous cycles (123–127). Clock^−/−^ mutant mice, for example, have extended and disrupted estrous cycles under both a light/dark cycle and during continuous darkness. In humans, it was reported that women with single-nucleotide polymorphisms in the ARNTL (*Bmal1*) have more miscarriages and less pregnancies than those without (128). Although these experiments have pointed to a crucial role of the SCN in the proper timing of estrous cyclicity, reproductive impairment following clock gene mutations could as well result from peripheral clock desynchronization (see Other Clocks in the Reproductive System). Interestingly, the reproductive phenotypes of young Clock-, Bmal1-, or Per1/Per2-mutated mice resemble that of middle-aged (over 10-month old) wild-type mice, with increased length and decreased frequency of estrous cycles (129, 130). These observations indicate that alterations in central or peripheral clocks may lead to advanced reproductive senescence (130).

The preovulatory LH surge is initiated by a SCN-derived stimulatory signal, at a time closely preceding general activity onset. However, this signal is effective at stimulating GnRH neurons to produce the LH surge only when estradiol concentrations have reached a critical threshold. Prior to the day of proestrus, the developing ovarian follicles secrete insufficient estradiol to fulfill this criteria and therefore, the SCN signal does not trigger the LH surge (56). The occurrence of the daily stimulatory SCN signal can be unmasked by implanting female rodents with estradiol capsules that result in proestrus concentrations of this hormone; in this case, a LH surge occurs every day (3, 56, 131).

#### SCN Neuropeptides Involved in the Timing of the GnRH/LH Surge

Transplant of fetal SCN tissue into bilaterally SCN-lesioned hamsters restores locomotor, but not endocrine rhythms in the absence of neural outgrowth, suggesting that intact neural connections are required for endocrine rhythmicity, whereas behavioral rhythms can be supported by a diffusible signal (132). Neuroanatomical studies have pointed to two putative SCN neural outputs signaling daily information to the reproductive axis, AVP and vasoactive intestinal peptide (VIP). Early experiments have identified SCN-originating, VIP-containing fibers contacting GnRH neurons (133, 134), which express the VIP receptor VPAC2 (135). However, more recent studies indicate that the SCN signals the time of day to GnRH neurons indirectly via AVP fibers projecting to the Kp neurons of the AVPV (78, 89).

Anterograde tracing studies show that a number of AVP-containing axons originating in the SCN make appositions to Kp neurons, whereas very few or no VIP terminals were found apposed to Kp neurons (87, 89). Furthermore, AVP is released with a peak coinciding with the onset of the LH surge (136) and AVPV Kp neurons express V1a receptors (87). Interestingly, the AVP input to Kp neurons is sensitive to estradiol since estradiol treatment significantly increases the number of AVP terminal appositions on individual Kp neurons (89) and circadian expression of V1a mRNA is abolished in ovariectomized animals (88). Furthermore, AVP signaling onto Kp neurons is critically dependent on circulating estradiol as AVP no longer activates Kp neurons in ovariectomized mice, an effect that is fully restored by estradiol treatment (90). Altogether, these results are consistent with the hypothesis that Kp neurons located in the rodent AVPV receive daily information from the SCN via an AVPergic monosynaptic pathway, a signal which is modulated (gated) by circulating estradiol (Figure 1A).

In line with these neuroanatomical observations, earlier physiological experiments pointed to a functional role of AVP in the GnRH/LH surge timing, even though the importance of Kp neurons was not yet known. Inhibition of AVP signaling with a V1a antagonist resulted in a reduction in the estradiol-induced LH surge (137), while intracerebroventricular infusion of AVP in SCN-lesioned, ovarectomized, and estradiol-treated rats was able to induce an LH surge (138). Furthermore, in co-cultures of POA and SCN, the GnRH surge was coordinated with the rhythm in AVP, but not VIP, and administration of AVP, not VIP, to preoptic explants in the presence of estradiol significantly increased GnRH release, providing further evidence for an important role of AVP in the LH surge generation (139). Finally, a recent study reported that intracerebroventricular administration of AVP in female Syrian hamsters activates Kp neurons similarly in the early or late part of the day, while in the same animals GnRH neurons are activated only late in the day (87). This observation was further confirmed by *in vitro* electrophysiological recordings of Kp-GFP neurons showing that AVP increases the firing rate of most Kp neurons during proestrus, independently of the time of day (90). Altogether these findings indicate that AVP activates Kp neurons every day, and the daytime gating of the GnRH/LH surge does not take place through SCN AVP–AVPV Kp signaling but rather downstream at the AVPV Kp–POA GnRH signaling.

A significant role of the SCN-derived VIP output in female reproduction should not be excluded (Figure 1A). Indeed, VIP afferents on GnRH neurons are sexually dimorphic, with female rats exhibiting higher VIPergic innervation than males (134). Furthermore, central administration of VIP antiserum reduces the LH surge (140), while central infusion of VIP is able to rescue the LH surge in middle-aged female rats (141). Finally, blocking the VPAC_2_ receptor attenuates GnRH neuronal cell firing during the afternoon surge in female, estradiol-treated mice (142). A recent study reported that the SCN-derived VIP neurons project to RFRP-3 neurons and central administration of VIP markedly suppresses RFRP-3 cellular activity in the evening, but not the morning, therefore indicating a specific role of VIP on neurons expressing RFRP-3, a neuropeptide thought to participate in the circadian-timed removal of estradiol negative feedback (143).

### Other clocks in the reproductive system

It has long been thought that the SCN-driven outputs are the sole source forwarding circadian signals to the female reproductive system. However, a growing body of evidence now suggests that structures and organs that are part of the gonadotropic axis might also play an intrinsic role in the timing of female reproduction.

In mammals, the timing system is now described as a multioscillator hierarchy of coordinated and synchronized cell and tissue clocks (144). The use of *Per1-luc* transgenic rats (145) and Per2:LUCIFERASE transgenic mice (115), where the *Per1* or *Per2* promoter drives expression of the *luciferase* gene, was decisive for the demonstration that non-SCN central structures and peripheral organs can sustain endogenous circadian oscillations. Thus, central structures (e.g., olfactory bulb, ARN, and retrochiasmatic area) and peripheral organs (e.g., liver, lung, heart, and kidney) are able to display endogenous-sustained circadian rhythmicity. The phase and the period of these “peripheral” clocks are tissue characteristic and different from those of the “central” clock of the SCN. The strength of their endogenous oscillations is often lesser than that of the SCN since according to tissues (and transgenic mice) the oscillations last from 2 to 20 cycles on average, whereas those of SCN can last several months. The oscillations generated by the peripheral clocks are independent of the SCN activity (they persist in SCN-lesioned animals) but their rhythms appear synchronized by the master clock, which is sometimes referred to as the conductor of the organism’s multi-oscillatory network. In the context of such a complex circadian network, recent evidence now suggests that the hypothalamo-pituitary gonadal axis is also a functional multi-oscillatory axis. Indeed, reproductive tissues from hypothalamic Kp and GnRH neurons down to the ovaries and the uterus display endogenous circadian oscillations of clock genes, as explained below. However, the functional role of these reproductive clocks with regards to the timing of reproduction (ovulation, implantation, and parturition) has yet to be determined.

#### Kisspeptin Neurons

Daily and circadian activities of Kp neurons in the AVPV area have been investigated in female rodents because of their strategic position between the integration of SCN-derived AVP input on one hand (87, 89) and the triggering of the preovulatory GnRH/LH surge on the other (19). Additionally, the modulatory effect of circulating estradiol on the daily activity of Kp neurons has also been investigated because of the potent effect of estradiol on Kp synthesis (77). Under high circulating estradiol levels, either in proestrus or in ovariectomized + estradiol-supplemented rodents, Kp neuronal activity (as seen by c-FOS activation) and Kiss1 mRNA are significantly increased about 3 h before lights off, thus 2 h before the LH surge (9, 86–88). Furthermore, we recently reported that Kp immunoreactivity is markedly but transiently decreased at the same time (9). In contrast, in low circulating estradiol conditions, in diestrus or ovariectomized animals, the daily variation in neuronal activity, Kiss1 mRNA and Kp immunoreactivty is abolished or strongly dampened (9, 86, 88). The daily activation of Kp neurons is triggered by the SCN AVP input since central injection of AVP induces c-FOS in Kp neurons and increases Kiss1 mRNA (87). Although AVP is released from the SCN neurons in the afternoon (146), AVP can activate Kp neurons in the morning or in the afternoon, indicating that the daily control of the LH surge is not gated by the AVPV Kp neurons (87). A recent study recording Kp neuron electrical activity confirmed that Kp neuron responsiveness to AVP depends on the concentrations of estradiol (90). Altogether these data indicate that under high circulating estradiol (when oocytes are mature enough to be released), Kp neurons can be activated by the SCN-derived AVP to increase Kp synthesis and release in order to induce GnRH neuronal activation and the downstream LH surge. In addition to the AVP activation of Kp neurons, we recently reported that these neurons host an intrinsic circadian clock, named Kiss-Clock (9). A preliminary study reported that the clock genes *Per1* and *Bmal1* are expressed in the rat AVPV, but their cellular localization was not established (88). We further demonstrated that virtually all AVPV Kp neurons express the PER1 protein (Figure 1B) with a daily rhythm both in proestrus and diestrus, but with a phase delay of about 3 h in diestrus as compared to proestrus (9). Furthermore, we reported that isolated Kp-expressing AVPV explants from PER2:LUCIFERASE mice display endogenous circadian oscillations with a period of 23.2 h (thus 1 h shorter than the SCN circadian period of the same mice), confirming the presence of an intrinsic circadian oscillator in AVPV Kp neurons (9) (Figure 1C). Remarkably, the period of this circadian clock is increased by 1 h in the presence of estradiol in the culture medium (Figure 1C), which is in line with the observed phase difference in PER1 expression according to the estrous stage. In contrast, the period of the SCN clock is not altered by environmental estradiol either *in vivo* or *in vitro* (9). The role of this Kiss-Clock has yet to be established. However, according to previous studies one might hypothesize that it could time sensitivity to estradiol since *ER*α** gene expression can be regulated by the BMAL1/CLOCK dimer (147), or it could also impact *Kiss1* gene expression since a circadian transcriptional factor, albumin D-site binding protein (Dbp), was reported to trigger *Kiss1* transcription via the D-box (148).

#### GnRH Neurons

Because of their pulsatile activity and critical role in timing the LH surge, GnRH neurons were the first in the reproductive system to be reported to express clock genes and display circadian activity. Indeed, all core clock genes (*Clock*, *Bmal1*, *Per1/2*, and *Cry1/2*) are expressed and cycle with a circadian period in both GT1-7 GnRH neuronal cell lines and GnRH neurons (149–152). Disruption of the circadian clock by transient expression of Clock Δ 19 in GT1-7 cells decreases the GnRH pulse frequency, while overexpression of *Cry1* in the same cells increases GnRH pulse amplitude (149). The GnRH clock could also regulate the timing of the neuronal sensitivity to upstream inputs. Indeed, the ability of VIP to activate GnRH neurons depends on the time of day and the estradiol environment (153), and the sensitivity of GT1-7 cells to release GnRH upon Kp or VIP treatment is time dependent (151). This time-dependent sensitivity may explain why central infusion of Kp fails to advance the onset of the LH surge in either naturally cycling or ovariectomized estradiol-supplemented female rodents (154, 155). Finally, the stimulatory and synchronizing effects of Kp on GnRH release are reduced in preoptic explants of *Bmal1* KO mice (35). Therefore, the circadian clock in GnRH neurons may provide a time-keeping mechanism to appropriately release GnRH under Kp, and possibly VIP, stimulation.

#### Pituitary Gland and Gonadotroph Cells

The pituitary, as a whole, was among the first peripheral oscillators found to display strong sustained circadian oscillations with a circadian period of about 23.8 h in *Per1*- or PER2-luciferase transgenic rodents (115, 145). It was further established that all clock genes expressed a daily rhythm in the whole pituitary but with a different profile according to the estrus stage (127, 156). The pituitary gland is made of different cell types, which could host several circadian oscillators with different phases. Expression of all cognate clock genes was identified in the alphaT3-1 gonadotroph cell line (147) and GnRH activation was reported to selectively increase *mPer1* expression in gonadotroph cells (157). Furthermore, the gene coding for the GnRH receptor contains non-canonical E-box promoter elements and *Bmal1* knockdown in a gonadotrope cell line reduces GnRH receptor mRNA (147). These studies raised the hypothesis that an intrinsic clock in gonadotrophs could directly regulate GnRH signaling and LH surge timing. To test this hypothesis, a specific BMAL1 KO disruption was performed in the gonadotrophs (127). The mutated mice still displayed a preovulatory LH surge and estrous cyclicity (although with a significant increase in cycle length variance) and the average time of puberty and fertility performance was not altered. Taken together, these data suggest that the intrinsic clock in gonadotroph cells is dispensable for LH surge regulation but contributes to estrous cycle robustness (127).

#### Ovaries

The ovarian circadian clock is very well documented in many mammalian and non-mammalian species and its function has been thoroughly investigated both *in vivo* and *in vitro* (158–161). Each cell type of the ovary, including theca cells, granulosa cells, and oocytes have a circadian clock (162). Further analyses reported that clock gene rhythms are only observed in mature granulosa and luteal cells, indicating that these rhythms are activated at a specific stage of follicle development, possibly under the control of FSH acting as a synchronizer of follicular cell activities (163). Ovarian physiology is strongly regulated by gonadotropins, and current studies indicate that LH stimulates various clock genes in the ovaries (159, 164). Furthermore, the endogenous rhythm of Per-driven oscillations in isolated ovaries is significantly shifted by LH and FSH indicating that the ovarian circadian clock is entrained by hormonal signals from the pituitary (160). Recently, a study reported that mice with a conditional KO of *Bmal1* in steroidogenic cells show severe deficits in implantation success and compromised progesterone secretion (165). A previous study demonstrated a circadian rhythm in ovarian sensitivity to LH with a greater ovarian response at night as compared to day, indicating that the ovarian circadian clock may set its responsiveness to the LH surge (161). Finally, various ovarian genes, including those encoding for the LH receptor and enzymes involved in steroid hormone biosynthesis, display circadian rhythms in granulosa cells and these rhythms are altered following the silencing of *Bmal1* expression (163). Altogether, these findings indicate that the clock in the ovary may be involved in the timing of ovulation, steroid hormone synthesis, and follicular growth and differentiation.

#### Uterus and Oviduct

Global knockout of the *Bmal1* or *Clock* gene disrupts implantation, increases fetal reabsorption during pregnancy, and leads to a high rate of full-term pregnancy failures (124, 166). Early in 2002, clock genes were found to be expressed in the uterus and oviduct of mice (167). Furthermore, the oviduct was reported to display a daily rhythm of several clock genes and clock-controlled gene (168). In the uterus, the presence of sustained endogenous clock oscillations was demonstrated in tissue explants of PER2:LUCIFERASE mice (99). These uterine oscillations were sustained even during pregnancy suggesting that embryos may be submitted to the maternal clock *in utero* (169). Interestingly, the period of the uterine clock oscillations changes according to the estrous stage and it is decreased when the tissue is incubated with estradiol (99). Additionally, a targeted deletion of *Bmal1* in the myometrium indicates a role for myometrial *Bmal1* in maintaining normal timing of parturition (170). Although additional studies are required to determine the physiological role of the uterine and oviduct clocks, the data obtained so far suggest that the developing embryo may be subjected to rhythmic changes in the oviduct during transit to the uterus and in the uterus during pregnancy.

## Shift-Work Consequences on Reproductive Cycles and Fertility

### Shift work

The modern 24-h-functioning society requires that an increasing number of employees work outside of the natural active period, in shifted conditions. According to the International Labor Organization (ILO; 1990), working in shifts is “a method of organization of working time in which workers succeed one another at the workplace so that the establishment can operate longer than the hours of work of individual workers” at different daily and night hours. Under a fixed-shift system, working time can be organized in two or three shifts: the early, late, and/or nightshifts. Under a rotating-shift system, workers might be assigned to work shifts that vary regularly over time.

Over the last 20 years in United States, almost 27% of men and 16% of women experienced shift work (171). In 2012, 15% of French workers, including 9.3% women, were working under shift work either occasionally (8%) or permanently (7.4%). An increasing number of analyses report that alteration in working schedule is often associated with an increased risk of developing cardiovascular/metabolic/gastrointestinal disorders, some types of cancer, and mental disorders including depression and anxiety (172–174). Hence, in 2007, shift work was reclassified from a possible to a probable human carcinogen (class 2A) by the International Agency for Research on Cancer. A French law passed on December 20, 2014 listed shift work as a risk factor increasing professional arduousness.

Given the importance of the circadian systems in the regulation of female reproduction, and given the fetal exposure to the maternal daily rhythms in temperature, substrates, and hormones, female shift workers may display reproductive alterations, such as an increased risk of irregular menstrual cycles, endometriosis, miscarriage, low birth weight, or pre-term delivery (175–177). Such disturbances may result from altered SCN clock synchronization with rest-activity and feed-fast cycles and/or internal desynchronization amongst peripheral clocks, especially those of the reproductive axis. Indeed, a recent study reported that peripheral clock genes in lymphocytes of shift workers are strongly altered as compared to day workers (178). Additionally, recent animal studies have shown that the functioning of fetal clocks depends on maternal hormones (179) and possibly feeding and activity, then maternal circadian disruption during pregnancy may lead to fetal SCN and peripheral clocks desynchronization.

In addition to the effect of circadian dysregulation, it should be kept in mind that shift work-related alterations in other daily functions, particularly food intake and sleep, may indirectly impact female reproduction. Thus, obesity, which is often associated with shift work, has a strong impact on reproductive performance (180) and sleep disturbance in prepubertal girls can alter estradiol-dependent pubertal development (181).

### Modeling shift work in rodents

Shift work is a very complex situation and therefore, it is difficult to design animal model conditions that mimic human shift work. A recent review listed four relevant models that use altered timing of food intake, activity, sleep or light exposure, or a combination of several (182).

Very few *in vivo* animal studies have investigated the alteration of fertility or the LH surge after a shift in the light/dark cycle or a photoperiod change (6, 56, 183). In one study, female Syrian hamsters were submitted to a 3-h phase advance or delay (183). When the phase advance was applied between 1 and 3 days before estrous, the LH surge was not fully resynchronized to the new schedule, even after 3 days. However, when hamsters were submitted to a 3-h phase delay, the LH surge was synchronized to the dark onset more rapidly. Similarly, in ovariectomized transgenic GnRH-GFP mice, a dark phase advance led to an advance in the LH surge (56). When the photoperiod length was modified in female hamsters, the timing of the LH surge was shifted in a similar manner to the nocturnal onset of locomotor activity (6).

In mice, exposure to either phase advances or delays at the beginning and throughout pregnancy leads to a significant decrease in pregnancy success (184). Interestingly, an *in vitro* study analyzed the effect of a 6-h phase advance on endogenous circadian oscillation of the SCN and various peripheral clocks and found that the ovarian clock was not fully resynchronized 6 days after the phase shift (185).

Although these few studies indicate that shifts in light/dark cycle alter the timing of the preovulatory LH surge and the synchrony amongst reproductive clocks, it is obvious that new animal studies have to be developed in order to understand the mechanisms underlying the various effect of shift work on reproduction and fertility in females.

### Reproductive consequence in women under shift work

A number of studies have investigated the relationship between fertility and shift work or night work in women working in pharmaceutical industries, hospitals, slaughter houses, and canneries (175, 178, 186–200). Although human studies are limited in their use for understanding causality and underlying mechanisms of health consequences of shift work, most of the above studies have reported a negative impact of shift work on fertility. However, there is a large heterogeneity among these analyses especially regarding the fertility criteria examined: body temperature curve, menstrual disorders, time to get pregnant, etc. Furthermore, it is important to stress that clinical or biological criteria can be misinterpreted since irregular cycles, as an indicator of the reproductive axis sensibility to shift work, may have no correlation with subfertility and pregnancy capacity (197).

Using body temperature curves to follow menstrual cycles, shift work was found to be associated with higher rates of short cycles and inadequate luteal phases (188). Furthermore, a higher prevalence of menstrual disorders is often found in female shift workers as compared to the non-shift workers. For example, in the most relevant studies including the largest populations, irregular cycles are reported in 12–20% of shift workers and 7–10% in non-shift workers (175, 192). In a large Danish population (17,531 daytime workers and 3,907 shift workers), it was reported that fixed evening and fixed night female workers took longer to get pregnant with adjusted odds ratio around 0.80, compared to daytime workers, but there was no unequivocal evidence of a causal association between shift work and subfecundity since this reduction may be mediated by pregnancy planning bias or differential options for sexual contacts (187). Only a few studies have examined reproductive hormones with various conclusions. It has been reported that FSH and LH levels are not different between shift and day workers (190, 192), but a single measurement of LH without a gynecologic examination and cycle characterization is difficult to interpret. The levels of 17-β-estradiol were found to be significantly increased (178, 201) in female shift workers possibly due to a prolonged follicular phase (186). Some studies have reported no significant relationship between shift work and subfertility or dysmenorrhea (196–200). However, among these studies, one has only a few women included with a surprising 35% of control women displaying irregular cycles (196). Another study reported no significant subfertility in shift-working women 1 year after birth control termination but yet, these women displayed a delay to get pregnant twice as long compared to the day-working group (199).

The pineal hormone melatonin, whose nocturnal production is profoundly affected by shifts in light/dark conditions, has been proposed to display potential anti-estrogenic effects (202, 203). Urinary melatonin excretion tends to be lower with a delayed peak of production during shift work (190, 193, 201, 204). The combination of inhibition on melatonin secretion with estradiol mistiming has been proposed to be involved in the hormone-related cancers observed in night shift workers (204, 205).

The conflicting results regarding the negative effect of shift work on female reproduction probably reflect large differences in the shift-work schedules, duration, and age of exposure, with a high number of confounding factors (like stress, fatigue, obesity, etc.) as well as methodological limitations (206, 207). Despite these considerations, cycling disorders should be considered as a sensitivity or intolerance to shift work. Shift work during pregnancy has adverse effects including increased risk of miscarriage (208), although this is somewhat controversial (209). Yet, most authors recommend avoiding shift work during pregnancy.

## Conclusion

Daily and estrogenic regulations of female reproduction allow the timing of ovulation to coincide with optimal reproductive tract functioning (oocyte maturation and receptive reproductive tract), maximal arousal (general activity and sexual motivation), and the best environmental conditions (food resources and stress level). These general coordinations confer maximum adaptive advantage to insure the success of this high energy-demanding reproductive function. Although the central role of the master SCN clock in the daily regulation of the LH surge has been well documented, the recent evidence that other peripheral clocks are located all along the gonadotropic axis, from Kp and GnRH neurons to the ovaries and uterus, raises the question of their role in the timing of reproduction. The latest findings indicate that these local clocks may optimize circadian cell sensitivity to upstream signals and set appropriate timing of the downstream reproductive responses. Notably, application of phase shifts leads to different rates of clock resynchronization between the SCN and reproductive organs, suggesting internal desynchronization of the reproductive axis, as seen in other functional axes. In our current society, where a significant number of female workers are working night or evening shifts, the delicate timing of organization in the reproductive network can easily be disrupted. While numerous studies have reported negative consequences of shift work on metabolic and cardiovascular functions as well as cancer occurrence, there are surprisingly few epidemiologic studies in humans and mechanistic studies in animal models reporting the incidence of shift work on female fertility. Future studies in the field should, therefore, investigate the impact of daily rhythm alterations, as observed under shift-work conditions, jet lag, or sleep disturbance, on reproductive cycle and fertility both in animal models and humans.

## Author Contributions

VS and TB contributed equally to the writing of this review.

## Conflict of Interest Statement

The authors declare that the research was conducted in the absence of any commercial or financial relationships that could be construed as a potential conflict of interest.
